# Gardening and Healthy Ageing: A Study on Health Benefits

**DOI:** 10.1093/geroni/igaf122.1946

**Published:** 2025-12-31

**Authors:** Amir Chwa, Eliza Khoo, Sarah Goh, Donovan Lim, Chen Yunru, Jing Xuan Tan, Clement Chia, Cynthia Chen

**Affiliations:** National University of Singapore, Singapore, Singapore; National University of Singapore, Singapore, Singapore; National University of Singapore, Singapore, Singapore; National University of Singapore, Singapore, Singapore; National University of Singapore, Singapore, Singapore; National University of Singapore, Singapore, Singapore; National University of Singapore, Singapore, Singapore; National University of Singapore, Singapore, Singapore

## Abstract

This study explores the health benefits of gardening, particularly its relevance in an ageing population. As societies age, promoting sustainable and accessible activities that enhance physical and mental well-being is crucial. Gardening, a widely enjoyed activity, may offer significant health benefits. A cross-sectional study was conducted among 386 participants aged 30 to 99 years, assessing sociodemographic, gardening habits, and physical and mental health through a structured questionnaire. Participants were categorized into two groups based on gardening frequency: those who do not garden or garden occasionally and those who garden daily (26.4%). The primary motivation for gardening was “happiness or satisfaction”, while “insufficient time” was the most cited barrier. Analysis revealed that daily gardening was associated with 43% lower odds of poor health (OR = 0.57, 95% CI: 0.32-0.99, p = 0.0499), defined as having either anxiety, health limitations, or both. Although not statistically significant, results suggest daily gardening may also reduce the odds of anxiety and health limitations. These findings highlight gardening’s potential as a simple, cost-effective intervention for promoting healthy ageing. By fostering physical activity, relaxation, and social engagement, gardening could play a key role in enhancing overall well-being and improving quality of life. Given its accessibility, policymakers and healthcare professionals should consider incorporating gardening into public health initiatives. Future research could conduct a randomised controlled trial to evaluate the effectiveness and cost-effectiveness of gardening interventions, providing stronger evidence for its role in preventive health strategies for ageing populations.

